# Malarial Fever in England

**Published:** 1920-07-31

**Authors:** 


THE PUBLIC WELL-BEING—(*continued*)

### Malarial Fever in England.

There is a good deal of misconception in this country as to the incidence of malarial fever, and as to the extent of the danger of the spread and perpetuation of the disease in the British Isles as the result of the war. Let us therefore quote a few facts and figures in the briefest form, set out by Dr. Andrew Balfour, who speaks with sufficient authority, though he was giving what is called a "popular" lecture on western links, good and evil, with the tropics.

No less than 433 cases of malaria are known to have been contracted in England during the period between August 22, 1917, and the end of 1919, and of these it is instructive to note that a considerable proportion has been in children under fifteen years of age. The civil population has furnished 160 of these cases, the great majority of which have occurred in the county of Kent, chiefly in the islands of Sheppey and Grain, where the sources of infection include cases of true endemic malaria as well as cases of imported malaria. In 1919 there were 193 cases, and of these 75 occurred in the Kent islands, the remaining cases being widely scattered. Essex, Norfolk, Salop, Huntingdon, Northampton, Buckingham, and Hampshire were the counties in which most of the "sporadic" cases occurred. Some of the 433 cases have proved fatal, and there has been a considerable mortality among the malaria carriers themselves—so much so that the deaths from malaria have been from three or four times as numerous as they were in pre-war days.

Here, then, we have a disease link with the tropics which has been greatly strengthened by the war, and which it is to be feared will not be weakened for some time to come, though it is unlikely, considering the measures which have been taken for dealing with it, that it will assume a more important *rôle* than it possesses at present.

